# Modality-specific associations between sensory differences and autistic traits

**DOI:** 10.1177/13623613231154349

**Published:** 2023-02-19

**Authors:** Peter Bang, Kajsa Igelström

**Affiliations:** Linköping University, Sweden

**Keywords:** broad autism phenotype, central auditory processing disorder, dimensional perspective, pragmatic language, research domain criteria

## Abstract

Sensory processing differences measured by self- or parent-report co-segregate with quantitative autistic traits and have potential endophenotypic properties. It is not known to what extent this reflects generalized sensory dysfunction versus more specific associations involving individual senses or autistic trait domains. We combined Bayesian variable selection with dominance analysis to obtain a more nuanced understanding of modality-specific associations. We recruited two independent samples of adults to complete the Broad Autism Phenotype Questionnaire and the Glasgow Sensory Questionnaire. For each domain of autistic traits (social interaction, communication, cognitive rigidity), we performed stochastic search variable selection using Glasgow Sensory Questionnaire modality subscales as predictors while controlling for uncertainty in other variables. Dominance analysis was applied to the reduced models to evaluate the relative importance of predictors. Only auditory scores reliably predicted all three autistic traits when other modalities were accounted for. The proprioceptive scale, which included motor and interoceptive deficits, predicted communicative autistic traits more than other trait domains. The tactile scale appeared most specific for social autistic traits. Although the findings must be interpreted in light of the limitations of the questionnaires, the study suggests that auditory differences may be more likely than differences in other senses to be a robust sensory endophenotype relevant to autism.

**Lay abstract:**

Sensory symptoms are a major source of distress for many autistic people, causing anxiety, stress, and avoidance. Sensory problems are thought to be passed on genetically together with other autistic characteristics, such as social preferences. This means that people who report cognitive rigidity and autistic-like social function are more likely to suffer from sensory issues. We do not know what role the individual senses, such as vision, hearing, smell, or touch, play in this relationship, because sensory processing is generally measured with questionnaires that target general, multisensory issues. This study aimed to investigate the individual importance of the different senses (vision, hearing, touch, smell, taste, balance, and proprioception) in the correlation with autistic traits. To ensure the results were replicable, we repeated the experiment in two large groups of adults. The first group contained 40% autistic participants, whereas the second group resembled the general population. We found that problems with auditory processing were more strongly predictive of general autistic characteristics than were problems with the other senses. Problems with touch were specifically related to differences in social interaction, such as avoiding social settings. We also found a specific relationship between proprioceptive differences and autistic-like communication preferences. The sensory questionnaire had limited reliability, so our results may underestimate the contribution of some senses. With that reservation in mind, we conclude that auditory differences are dominant over other modalities in predicting genetically based autistic traits and may therefore be of special interest for further genetic and neurobiological studies.

## Background

Differences in sensory processing are prevalent in autism spectrum conditions (Ben-Sasson et al., 2019) and are often a cause of limitations in everyday situations (MacLennan et al., 2022). Sensory differences are heterogeneous, can include all senses, and may involve both over- and under-responsivity (Ben-Sasson et al., 2019). Sensory differences in autism have been linked to decreased social and adaptive abilities (Lane et al., 2010), repetitive behaviors (Scheerer et al., 2021), anxiety (Wigham et al., 2015), and an increased risk of coexisting mental health conditions compared to non-autistic peers (Leader et al., 2021; Rossow et al., 2021; Wang et al., 2019).

Sensory processing difficulties have been suggested to be a transdiagnostic phenotype associated with multiple psychiatric conditions (van den Boogert et al., 2022). There is a linear relationship between sensory differences and quantitative autistic traits (QATs) measured by the Autism Quotient (AQ) or the Broad Autism Phenotype Questionnaire (BAPQ) (Baron-Cohen et al., 2001; Horder et al., 2014; Hurley et al., 2007; Mayer, 2017; Robertson & Simmons, 2013; Sapey-Triomphe et al., 2018). Horder et al. (2014) found that this relationship remained after correcting for anxiety traits, migraine, and mental illness, suggesting it is somewhat specific for QATs. The association between QATs and sensory processing differences appears to be at least in part genetically based. A large twin study, using the Autism-Tics, attention deficit/hyperactivity disorder (ADHD), and other Comorbidities Inventory to measure both QATs and sensory reactivity, found that genetic factors explained the majority of the correlation between QATs and sensory scores across both clinical and non-clinical QAT ranges (Taylor et al., 2018). Neufeld et al. (2021) found a significant contribution of genetic factors to the associations between QATs (Social Responsiveness Scale) and scores on the Sensory Sensitivity subscale of the Adult/Adolescent Sensory Profile (AASP). Parents in multiplex autism families showed higher auditory and visual scores on the AASP compared to simplex families (Donaldson et al., 2017), possibly indicating a stronger genetic relationship between autism and these modalities. However, the polygenicity of autism and the approximately normal distribution of QATs and sensory scores (e.g. Robertson & Simmons, 2013) argue against a simple genetic explanation for sensory processing differences in autism.

Research on sensory differences in neurodevelopmental conditions has evolved out of early work in the field of occupational therapy (e.g. Ayres, 1972), which culminated in Winnie Dunn’s four-quadrant model of sensory processing (Dunn, 2007; Miller et al., 2007). In this model, sensory processing varies along two axes: neurological threshold and behavioral response. The axes cross to form four quadrants (low registration, sensory sensitivity, sensory avoiding, and sensory seeking), for which self-reported psychometric scores can be obtained with the Sensory Profile 2 or the AASP. Most previous studies on sensory differences in autism have used these instruments and have not considered individual sensory modalities (Ben-Sasson et al., 2019). However, sensory perception is a broad construct comprising multiple senses and levels of processing, and there is no a priori reason to assume that all sensory modalities contribute equally to the association with QATs.

The newer Glasgow Sensory Questionnaire (GSQ) contains modality-specific subscales, even though the internal reliabilities of the subscales are moderate to low (Kuiper et al., 2019; Robertson & Simmons, 2013). Nevertheless, elevated scores on the GSQ were reported in all sensory domains in participants with high QATs (Robertson & Simmons, 2013), and the AQ score correlated linearly with all seven modality-specific subscales on the GSQ (Sapey-Triomphe et al., 2018; Takayama et al., 2014). There is also some evidence that sensory difficulties cluster in ways that indicate modality-specific differences. For example, exploratory factor analysis of responses to the Short Sensory Profile in a large sample of autistic adults found separation of audiovisual, taste/smell, and tactile/movement sensitivity (Tomchek et al., 2014). Scheerer et al. (2021) also reported clustering of sensory patterns, separating individuals with taste/smell sensitivity from those with generalized differences in other senses.

If the sensory differences in autism are domain-general (i.e. solely contributable to a generalized mechanism), there should not be any substantial differences between modalities in their correlations with QATs. However, such comparisons are complicated because all GSQ and QAT constructs are highly collinear (Hurley et al., 2007; Robertson & Simmons, 2013; Sapey-Triomphe et al., 2018). This study aimed to enable comparisons between the sensory modalities through the use of Bayesian stochastic search variable selection (SSVS; Bainter et al., 2020) followed by dominance analysis (DA; Braun et al., 2019). The SSVS was used to identify modalities that reliably predicted QATs in the domains of (1) social interaction, (2) communication, and (3) rigid behaviors, and the DA was used to compare the relative importance of predictors.

## Methods

We used a dimensional transdiagnostic approach consistent with the Research Diagnostic Criteria (RDoC) framework (Cuthbert & Insel, 2013; Insel et al., 2010).

### Participants

In line with the recommendations to improve the replicability of clinical psychological science (Buxbaum et al., 2019; Tackett et al., 2017), we recruited two independent samples of adults to complete the GSQ and BAPQ: a discovery sample (*N* = 252) and a replication sample (*N* = 268). The participants were recruited using the platform Prolific.co, which is an online recruitment platform that gives access to a large and diverse population in terms of geographical location and ethnicity (Palan & Schitter, 2018). This allowed us to use the built-in prescreening filters to control sample composition without knowledge of the participants’ identities. All individuals reported English as their first language and resided in Australia, Canada, Ireland, New Zealand, the United Kingdom, or the United States. Data collection for the discovery sample was completed before starting recruitment for the replication sample, and there was no overlap between the two samples. Participants were reimbursed by Prolific for their participation through the built-in mechanism (researchers pay Prolific for the advertising, and Prolific pays participants). The study was exempt from ethical review according to the Swedish regulations because participants were fully anonymous to the researchers, and no personal data were collected. To protect anonymity, we limited the collection of demographic variables to those necessary for the analysis, which for the replication sample excluded socioeconomic status and educational attainment levels. The study was designed according to the principles of the Declaration of Helsinki. Participants provided digital informed consent and could exit the study at any point by closing the browser window.

The discovery sample was clinically enriched (40% autistic participants) as the data were collected as part of a different study that relied on group comparisons between neurotypical and autistic adults on the topic of repetitive behaviors (unpublished data). The approach ensured a broad distribution of QATs, with enough data in the highest ranges. The recruitment utilized two separate advertisements using non-overlapping Prolific filters (which determine which people can see the study): one was directed at people with an autism diagnosis according to their Prolific settings and one was aimed at everyone else. Exclusion criteria for the original study were intellectual disability, neurodegenerative conditions, psychotic conditions, tic and stereotypic movement conditions, and incomplete responses on psychometric scales. The advertisements linked to the same Qualtrics study, and autism status was determined based on the participants’ responses within our questionnaire. Given the anonymous nature of the study, we did not confirm diagnoses clinically.

Several studies have demonstrated a normal distribution of QATs in the general population, with no clear discontinuity between QATs and clinical autism (English et al., 2021; Ingersoll et al., 2011; Lundin et al., 2019; Sasson et al., 2013). Therefore, to extend the relevance of our findings, a second sample was recruited from the general population without selecting for clinical status, using a Prolific filter that excluded participants who had been part of the discovery sample. This resulted in a replication sample with 6% autistic participants. Exclusion criteria were psychotic illnesses, primary sensory issues, brain injury, neurodegenerative disease, and failure to pass attention checks. The clinical exclusion criteria were chosen due to their likely effects on sensory perception or self-report integrity. Attention checks were straightforward and consisted of regularly interspersed questions asking the participant to mark a certain alternative (e.g. “Are you paying attention? Please mark the alternative in the middle.”). While the use of attention checks might have excluded some participants with legitimate attention difficulties, we reasoned that any signs of inattention were associated with uncertain data integrity. Based on these criteria, we excluded 9 participants in the discovery sample and 33 participants in the replication sample. Demographic details are shown in Table 1.

**Table 1.** Demographic data.

	Discovery sample (*N* = 252)	Replication sample (*N* = 268)
Gender		
Woman	145	134
Man	91	126
Non-binary	13	7
Other/prefer not to say	3	1
Age (years ± SD)	34.1 ± 11.6	37.6 ± 12.6
Country of residence		
The United States	26 (10.3%)	81 (30.2%)
The United Kingdom	199 (79%)	150 (55.9%)
Australia	11 (4.4%)	9 (3.4%)
Canada	0 (0.0%)	21 (7.8%)
Ireland	9 (3.5%)	4 (1.4%)
New Zealand	7 (2.8%)	3 (1.1%)
Education		
Years of schooling ± SD	16.5 ± 3.9	–
Parents’ education		
Years of schooling ± SD	14.9 ± 4.7	–
Psychiatric conditions		
Autism spectrum conditions	101 (40.1%)	17 (6.3%)
Attention deficit/hyperactivity disorder	16 (6.4%)	27 (10.1%)
Developmental coordination disorder	20 (7.9%)	5 (1.9%)
Obsessive/compulsive disorder	22 (8.7%)	27 (10.1%)
Anxiety conditions	97 (38.4%)	140 (52.2%)
Mood conditions	77 (30.6%)	137 (51.1%)
Personality conditions	6 (2.3%)	19 (7.0%)

SD: standard deviation.

### Materials

#### The BAPQ

The BAPQ is a 36-item questionnaire developed to identify subclinical QATs in first-degree relatives of autistic children (Hurley et al., 2007). The BAPQ quantifies differences in social interaction, communication and cognitive rigidity, measured by the subscales aloof personality, pragmatic language deficits, and rigid personality, respectively. Items are in the format of statements such as “I enjoy being in social situations.” Each subscale consists of 12 items, and each item is rated on a 6-point Likert-type scale (1 = very rarely, 6 = very often). The scores were summed to derive total and subscale scores. In this study, the internal consistency was high for the total score and subscales (Cronbach’s α > 0.85; Table 2).

**Table 2.** Cronbach’s α for subscales of the BAPQ and GSQ.

Questionnaire/subscale	*N* items	Cronbach’s α (discovery sample)	Cronbach’s α (replication sample)
BAPQ	36	0.95	0.94
Aloof personality	12	0.92	0.94
Pragmatic language	12	0.85	0.85
Rigid personality	12	0.90	0.91
GSQ	40	0.92	0.90
Visual	6	0.70	0.69
Auditory	5	0.70	0.68
Tactile	6	0.64	0.54
Olfactory	6	0.49	0.52
Gustatory	5	0.65	0.54
Proprioception	6	0.72	0.62
Vestibular	6	0.66	0.61

BAPQ: Broad Autism Phenotype Questionnaire; GSQ: Glasgow Sensory Questionnaire.

The auditory and gustatory GSQ subscales contained five items due to the removal of items that overlapped with the rigid QAT construct (see section “Methods” for detail).

#### The GSQ

The GSQ was developed to measure sensory difficulties experienced by autistic people (Robertson & Simmons, 2013). The instrument contains 42 items covering all 7 sensory modalities (vision, hearing, taste, smell, touch, proprioception, vestibular). Items are scored on a 5-point Likert-type scale (1 = never, 5 = always). We used the original modality-specific subscales (summed scores) for group comparison (non-autistic vs autistic) and correlations with the total BAPQ score. For the main analysis, which tested relationships between sensory modalities and specific QATs, we removed two items from the GSQ that overlapped too much with the rigid subscale of the BAPQ. The removed items were “Do you like to listen to the same piece of music/part of a DVD over and over again?” (Auditory subscale) and “Do you eat the same foods most of the time?” (Gustatory subscale).

Items on the GSQ can also be divided into hyper- and hypo-sensitivity scales, where items probing hyposensitivity include sensory-seeking behaviors, non-responses to inputs, and difficulties deciphering inputs such as speech (Robertson & Simmons, 2013). As previously reported (Sapey-Triomphe et al., 2018), we found a strong positive correlation between the hyper- and hypo-sensitivity scores (*R*^2^ = 0.74, *p* < 0.001), supporting the pooling of scores within modalities. In both samples, the internal consistency was high for the total GSQ (Cronbach’s α > 0.90), whereas it was low to moderate for the subscales (Table 2), consistent with previous studies (Kuiper et al., 2019; Ujiie & Wakabayashi, 2015).

### Demographic questions

Demographic questions included age, country of residence, psychiatric conditions, and primary sensory deficits. The gender question had female and male options, as well as “non-binary” and “other/prefer not to say.” Dummy-coded gender variables were created for non-binary and male gender for use in analyses. We did not control for “other/prefer not to say” as only four participants chose this option (discovery sample, *N* = 3; replication sample, *N* = 1).

The discovery sample specified psychiatric conditions in a matrix-style question, with one condition per row. Participants who answered “Yes” to having been diagnosed with autism were directed to follow-up questions about the age of diagnosis and the name of the condition (not reported in this study but used for quality control). The replication sample answered Yes/No to a similar but less extensive list of conditions (excluding stereotypic movement condition and non-epileptic seizures). For both samples, the question about autism diagnosis had an additional middle option of “No, but I/someone suspects it” (the discovery sample) or “self-identify, self-diagnosed or under evaluation” (the replication sample) to acknowledge self-diagnosis without conflating it with clinically diagnosed autism.

### Statistical analysis

We tested for normal distributions using the Shapiro–Wilk tests. To explore which sensory modalities may be associated with higher QATs, we used SSVS (Bainter et al., 2020). SSVS is a Bayesian framework used for empirically driven variable selection (George & Mcculloch, 1993). The SSVS uses the Markov chain Monte Carlo sampling to sample from a posterior distribution of the possible subsets of predictors to identify the best models. Predictors selected more frequently in the sampling receive higher marginal inclusion probabilities (MIPs; 0.0–1.0) (Bainter et al., 2020; George & Mcculloch, 1993). This approach selects predictors while controlling for uncertainty in other predictors included in the model, maximizing power and minimizing false positives.

For each sample, three analyses were performed using an online application that performs SSVS (Bainter et al., 2020; https://ssvsforpsych.shinyapps.io/ssvsforpsych/). All analyses used the following SSVS specifications: prior inclusion probability α = 0.5 (indicating that each predictor has a 50/50 prior probability of being included in the model), 5000 burn-in iterations to achieve convergence, and 20,000 total iterations. The seven modality-specific GSQ subscores, age, ADHD, developmental coordination disorder (DCD), anxiety conditions, mood conditions, and the two dummy-coded gender variables (woman = 0) were entered as independent variables, with one of the QATs (social interaction, communication, or cognitive rigidity) as the dependent variable. To assess convergence and ensure that SSVS results were stable, we ran SSVS analyses twice and computed a Pearson’s correlation between each variable’s estimated MIPs. The obtained correlation exceeded *R* = 0.99 for all analyses.

We applied DA to each of the models returned by the SSVS using the procedure described in Braun et al. (2019) and applied in White et al. (2022). We calculated general dominance weights by computing each predictor’s averaged incremental validity across all possible subset regression models involving that predictor over 1000 Monte Carlo simulated runs (Azen & Budescu, 2003; Braun et al., 2019). This method addresses the issue of sampling error variance that impacts individual instances of DA weights (Braun et al., 2019). We did not correct for measurement error (Braun et al., 2019) because the low subscale reliabilities of the GSQ attenuated inter-variable correlations and made the correlation matrices non-positive definite (Johnson, 2004).

### Community involvement

Community members were not involved in the study.

## Results

### Replication of sensory differences in participants with high autistic traits

The discovery sample was used to replicate previously observed sensory processing differences between autistic and non-autistic adults. The total GSQ and all modality subscales were significantly elevated in autistic participants, and there were significant bivariate correlations between the BAPQ and all GSQ scores (Table 3). BAPQ–GSQ correlations were significant also when autistic individuals were excluded (*N* = 151, data not shown).

**Table 3.** Replication of group differences and bivariate relationships (discovery sample).

GSQ modality	Non-autistic (median ± SD)	Autistic (median ± SD)	Mann–Whitney test	Spearman’s correlation with BAPQ
Total scale	94 ± 17	114 ± 21	*U* = 3130.0, *p* = 2.2 × 10^−15^	ρ = 0.583, *p* = 2.3 × 10^−24^
Visual	13 ± 3.4	16 ± 4.1	*U* = 4446.5, *p* = 1.9 × 10^−8^	ρ = 0.442, *p* = 1.7 × 10^−13^
Auditory	18 ± 3.5	22 ± 4.0	*U* = 3672.0, *p* = 2.7 × 10^−12^	ρ = 0.546, *p* = 5.3 × 10^−21^
Tactile	12 ± 3.8	16 ± 4.2	*U* = 4254.0, *p* = 2.5 × 10^−9^	ρ = 0.459, *p* = 1.5 × 10^−14^
Olfactory	13 ± 2.8	15 ± 3.6	*U* = 4938.5, *p* = 1.9 × 10^−6^	ρ = 0.329, *p* = 8.7 × 10^−8^
Gustatory	13 ± 3.5	16 ± 3.6	*U* = 4335.5, *p* = 5.9 × 10^−9^	ρ = 0.452, *p* = 4.3 × 10^−14^
Proprioceptive	11 ± 3.0	15 ± 4.2	*U* = 3050.5, *p* = 5.6 × 10^−16^	ρ = 0.519, *p* = 8.9 × 10^−19^
Vestibular	12 ± 3.0	15 ± 4.3	*U* = 4215.0, *p* = 1.6 × 10^−9^	ρ = 0.451, *p* = 5.0 × 10^−14^

BAPQ: Broad Autism Phenotype Questionnaire; GSQ: Glasgow Sensory Questionnaire; SD: standard deviation.

### Auditory and tactile differences dominate as predictors of social QATs

**Figure 1.**
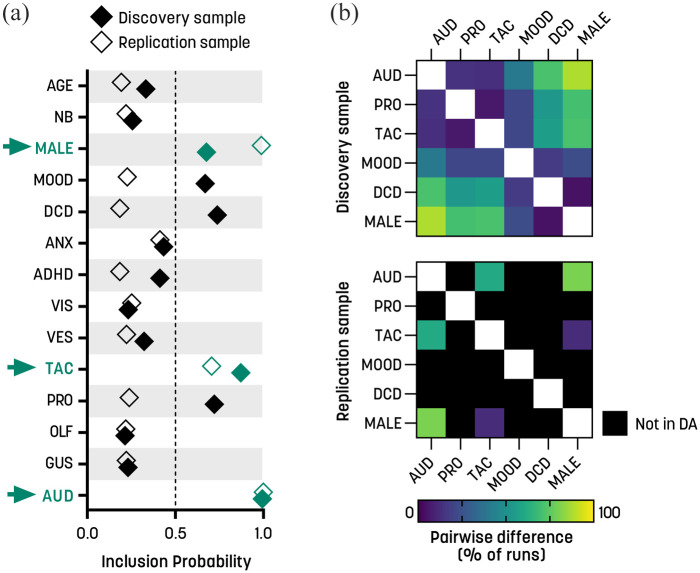
Social QATs: stochastic search variable selection (SSVS) and dominance analysis. (a) Marginal inclusion probabilities for the sensory subscales and covariates for the two independent samples. The arrows highlight the predictors that exceeded the inclusion threshold of 0.5 in both samples. (b) Pairwise differences between predictors. The symmetric matrices illustrate the percentage of times that pairs of predictors were significantly different from each other in the dominance analysis, in the discovery sample (upper panel) and replication sample (lower panel). Black squares indicate that the predictor was not included in the dominance analysis due to not being selected by the SSVS (see section “Methods” for details). ANX: anxiety conditions; ADHD: attention deficit/hyperactivity disorder; AUD: auditory; DCD: developmental coordination disorder; GUS: gustatory; MOOD: mood conditions; NB: non-binary gender; OLF: olfactory; PRO: proprioceptive; TAC: tactile; VES: vestibular; VIS, visual.

When social QATs were used as the dependent variable, the SSVS found MIPs nearing 1.0 for the auditory modality in both the discovery sample and the replication sample (Figure 1(a)). Tactile differences along with the male gender variable also showed a high MIP across both samples (Figure 1(a)). In the discovery sample, the SSVS additionally selected DCD, mood conditions, and the proprioceptive modality (filled diamonds in Figure 1(a)). The SSVS identifies robust predictors but does not test their relative importance. Therefore, we ran DAs on models including the selected predictors. The DA for the discovery sample included male gender, DCD, and mood conditions along with auditory, tactile, and proprioceptive scores. The DA for the replication sample included male gender, tactile, and auditory scores. The auditory modality demonstrated the largest averaged weight, highest averaged rank, and the largest proportion of times significantly different from zero in both analyses (Table 4). This result was most robust in the replication sample, where the auditory predictor showed the highest rank (Table 4) and was significantly different from tactile scores and male gender in 60% and 80% of runs, respectively (Figure 1(b), lower panel). In the discovery sample, the auditory, tactile, and proprioceptive predictors demonstrated approximately equal weights, ranks, and significance values (Table 4), and were statistically distinct from each other in less than 14% of runs (Figure 1(b), upper panel). Overall, the sensory predictors appeared to be more robust than the non-sensory predictors (Table 4 and Figure 1(b)).

**Table 4.** General dominance analysis weight and rank values for predictors of social QATs in the discovery sample and the replication sample.

Predictors	Discovery sample		Replication sample	
	M [95% CI]	Sig	M [95% CI]	Sig
Weights				
Auditory	0.09 [0.04, 0.16]	95%	0.14 [0.07, 0.21]	94%
Tactile	0.07 [0.03, 0.12]	87%	0.05 [0.01, 0.11]	32%
Proprioception	0.07 [0.03, 0.11]	89%	–	–
DCD	0.02 [0.00, 0.06]	7%	–	–
Mood conditions	0.04 [0.01, 0.09]	32%	–	–
Male	0.01 [0.00, 0.03]	1%	0.03 [0.00, 0.07]	9%
*R*^2^	0.30 [0.21, 0.39]		0.22 [0.14, 0.32]	
Ranks				
Auditory	1.55 [1.00, 4.00]		1.03 [1.00, 2.00]	
Tactile	2.38 [1.00, 4.00]		2.29 [2.00, 4.00]	
Proprioception	2.56 [1.00, 4.00]		–	
DCD	5.09 [3.00, 7.00]		–	
Mood conditions	3.93 [2.00, 6.00]		–	
Male	5.78 [4.00, 7.00]		2.96 [2.00, 4.00]	

QAT: quantitative autistic trait; DCD: developmental coordination disorder; M: mean; CI: confidence interval; Sig: proportion of runs that the predictor was found to be significantly different from zero (i.e. the spurious predictor).

The spurious predictor used to test for significant differences from zero was excluded from the table. Mean represents the average value across all simulated runs, and all values were based on 1000 simulated runs.

### Auditory and proprioceptive differences dominate as predictors of communicative QATs

**Figure 2.**
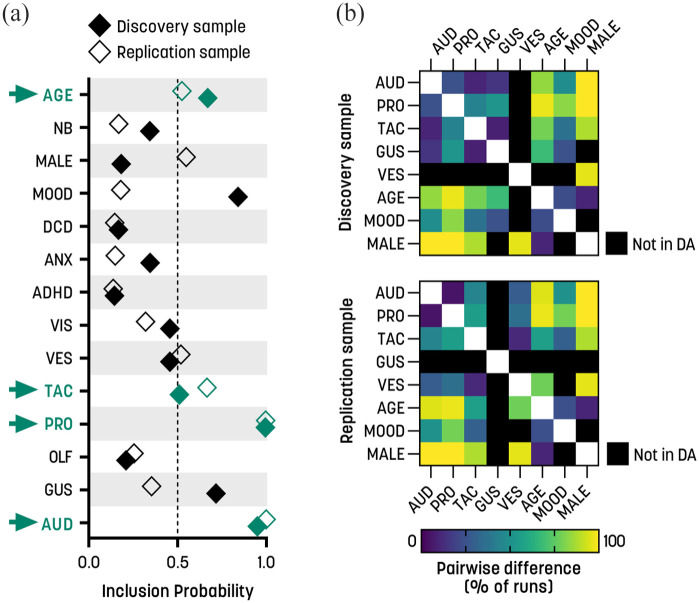
Communicative QATs: stochastic search variable selection (SSVS) and dominance analysis. (a) Marginal inclusion probabilities for the sensory subscales and covariates for the two independent samples. The arrows highlight the predictors that exceeded the inclusion threshold of 0.5 in both samples. (b) Pairwise differences between predictors. The symmetric matrices illustrate the percentage of times that pairs of predictors were significantly different from each other in the dominance analysis, in the discovery sample (upper panel) and replication sample (lower panel). Black squares indicate that the predictor was not included in the dominance analysis due to not being selected by the SSVS (see section “Methods” for details). ANX: anxiety conditions; ADHD: attention deficit/hyperactivity disorder; AUD: auditory; DCD: developmental coordination disorder; GUS: gustatory; MOOD: mood conditions; NB: non-binary gender; OLF: olfactory; PRO: proprioceptive; TAC: tactile; VES: vestibular; VIS: visual.

For communicative QAT scores, the auditory and proprioceptive modalities showed very high MIPs in the SSVS (Figure 2(a)). The tactile modality and age also exceeded the MIP cut-off of 0.5 in both samples, but less robustly than the auditory and proprioceptive scores (Figure 2(a)). In the discovery sample, mood conditions and gustatory scores were also selected, and in the replication sample, the vestibular scores and male gender were selected. The proprioceptive and auditory modalities showed general dominance in the DA in both samples (Table 5). Pairwise comparisons showed that the two modalities were significantly different from each other in only 20% (discovery sample) and 5% (replication sample) of runs (Figure 2(b)), indicating no obvious dominance of one over the other. The tactile predictor was significantly different from the proprioceptive and auditory modalities in 11%–55% of runs (Figure 2(b)). Vestibular and gustatory scores explained intermediate proportions of variance (Table 5) and differed from the auditory and proprioceptive modalities in 29%–49% of runs (Figure 2(b)). Mood conditions explained a small amount of variance in the discovery sample (Table 5), and age and gender were unimportant in both samples and differed from the sensory scores in most runs (Table 5 and Figure 2(b)).

**Table 5.** General dominance analysis weight and rank values for predictors of communicative QATs in the discovery sample and the replication sample.

Predictors	Discovery sample		Replication sample	
	M [95% CI]	Sig	M [95% CI]	Sig
Weights				
Proprioception	0.15 [0.10, 0.20]	100%	0.13 [0.08, 0.19]	100%
Auditory	0.11 [0.06, 0.16]	100%	0.13 [0.08, 0.19]	100%
Tactile	0.09 [0.06, 0.13]	100%	0.07 [0.03, 0.12]	96%
Vestibular	–	–	0.08 [0.05, 0.13]	100%
Gustatory	0.08 [0.04, 0.13]	99%	–	–
Age	0.02 [0.00, 0.6]	18%	0.02 [0.00, 0.05]	18%
Mood conditions	0.05 [0.02, 0.09]	72%	–	–
Male	–	–	0.01 [0.00, 0.03]	1%
*R*^2^	0.50 [0.41, 0.58]		0.45 [0.34, 0.56]	
Ranks				
Proprioception	1.24 [1.00, 3.00]		1.58 [1.00, 3.00]	
Auditory	2.47 [1.00, 4.00]		1.60 [1.00, 3.00]	
Tactile	3.09 [2.00, 5.00]		3.64 [2.00, 5.00]	
Vestibular	–		3.21 [2.00, 4.00]	
Gustatory	3.48 [1.00, 5.00]		–	
Age	5.89 [5.00, 7.00]		5.14 [4.00, 6.00]	
Mood conditions	4.89 [3.00, 6.00]		–	
Male	–		6.04 [5.00, 7.00]	

QAT: quantitative autistic trait; M: mean; CI: confidence interval; Sig: proportion of runs that the predictor was found to be significantly different from zero (i.e. the spurious predictor).

The spurious predictor used to test for significant differences from zero was excluded from the table. Mean represents the average value across all simulated runs, and all values were based on 1000 simulated runs.

### Auditory differences dominate in predicting rigid QATs

**Figure 3.**
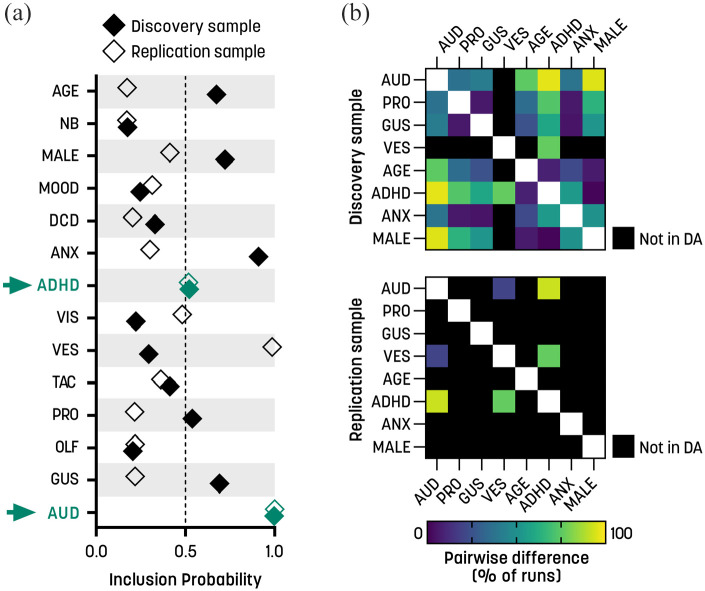
Rigid QATs: stochastic search variable selection (SSVS) and dominance analysis. (a) Marginal inclusion probabilities for the sensory subscales and covariates for the two independent samples. The arrows highlight the predictors that exceeded the inclusion threshold of 0.5 in both samples. (b) Pairwise differences between predictors. The symmetric matrices illustrate the percentage of times that pairs of predictors were significantly different from each other in the dominance analysis, in the discovery sample (upper panel) and replication sample (lower panel). Black squares indicate that the predictor was not included in the dominance analysis due to not being selected by the SSVS (see section “Methods” for details). ANX: anxiety conditions; ADHD: attention deficit/hyperactivity disorder; AUD: auditory; DCD: developmental coordination disorder; GUS: gustatory; MOOD: mood conditions; NB: non-binary gender; OLF: olfactory; PRO: proprioceptive; TAC: tactile; VES: vestibular; VIS: visual.

The auditory modality and ADHD were selected as predictors of rigid QATs in both samples (Figure 3(a)). In the discovery sample, the SSVS also returned MIPs above the threshold for the proprioceptive and gustatory modalities, along with age, maleness, and anxiety conditions. In the replication sample, the vestibular score was selected instead (Figure 3(a)). The auditory modality showed general dominance over ADHD in both samples (Table 6). In the discovery sample, the proprioceptive score, gustatory score, and anxiety explained an equal amount of variance but were significant in a smaller proportion of runs than the auditory modality (Table 6). In the replication sample, the vestibular modality explained almost as much variance as the auditory modality (Table 6), and the auditory scores were significantly different from vestibular scores in 20% of runs (Figure 3(b)). Across both samples, the demographic variables age, ADHD, and maleness were unimportant and differed from the dominant predictor in >75% of cases (Figure 3(b)).

**Table 6.** General dominance analysis weight and rank values for predictors of rigid personality in the discovery sample and the replication sample.

Predictors	Discovery sample		Replication sample	
	M [95% CI]	Sig	M [95% CI]	Sig
Weights				
Proprioception	0.06 [0.03, 0.11]	73%	–	–
Auditory	0.11 [0.06, 0.18]	95%	0.13 [0.07, 0.20]	96%
Vestibular	–	–	0.09 [0.04, 0.15]	79%
Gustatory	0.06 [0.02, 0.11]	61%	–	–
Age	0.03 [0.00, 0.07]	12%	–	
Anxiety conditions	0.06 [0.02, 0.10]	55%	–	–
AD/HD	0.01 [0.00, 0.03]	1%	0.01 [0.00, 0.05]	3%
Male	0.01 [0.00, 0.03]	2%	–	–
*R*^2^	0.34 [0.24, 0.45]		0.24 [0.15, 0.34]	
Ranks				
Proprioception	2.88 [1.00, 5.00]		–	
Auditory	1.19 [1.00, 3.00]		1.16 [1.00, 2.00]	
Vestibular	–		1.85 [1.00, 2.00]	
Gustatory	3.22 [1.00, 5.00]		–	
Age	5.23 [3.00, 8.00]		–	
Anxiety conditions	3.15 [1.00, 5.00]		–	
AD/HD	6.74 [5.00, 8.00]		3.43 [3.00, 4.00]	
Male	6.46 [4.00, 8.00]	–	–	

AD/HD: attention deficit/hyperactivity disorder; M: mean; CI: confidence interval; Sig: proportion of runs that the predictor was found to be significantly different from zero (i.e. the spurious predictor).

The spurious predictor used to test for significant differences from zero was excluded from the table. Mean represents the average value across all simulated runs, and all values were based on 1000 simulated runs.

## Discussion

This study investigated the relative importance of modality-specific sensory difficulties in predicting social, communicative, and rigid QATs in an adult population. High scores on the auditory modality subscale were strongly predictive of traits in all three QAT domains, suggesting that auditory processing differences may be a robust endophenotype that co-segregates with the broad autism phenotype. In contrast, more specific associations were seen for the tactile and proprioceptive modalities, which predicted social and communicative QATs, respectively. The absence of robust associations for olfactory, gustatory, vestibular, and visual modalities must be interpreted in light of the moderate internal reliabilities of the GSQ subscales, which risk attenuating correlations (Table 2). However, it might suggest that deficits in these modalities are inherited differently or are associated more specifically with autism as a diagnostic category rather than QATs. Furthermore, we reproduced patterns of gender differences in social QAT (Hurley et al., 2007; Nayar et al., 2021; Sasson et al., 2013), age differences in communication QAT (Chopik et al., 2021; Tillmann et al., 2018), and fewer rigid QATs in ADHD (Johnston et al., 2011; Panagiotidi et al., 2019; Polderman et al., 2013).

The dominance of the auditory modality is consistent with the known presence of sound sensitivity in autistic populations and first-degree relatives (Donaldson et al., 2017; Sapey-Triomphe et al., 2018; Strömberg et al., 2022), but it does not give any mechanistic explanation. The GSQ auditory subscale probes multiple auditory functions, including aversion to specific, loud, or unpredictable sounds, attraction to specific sounds, and difficulties with speech perception (Robertson & Simmons, 2013). These difficulties are prevalent in autism but are likely to engage disparate mechanisms, such as auditory brainstem abnormalities, sensory gating deficits, disturbed central gain control, or broader networks involved in emotional reactions to sounds. Subjective auditory sensitivity did not correspond to altered thresholds for detection or discrimination (Schulz & Stevenson, 2022; Yaguchi & Hidaka, 2020), but the behavioral threshold for discomfort or startle is often lowered in autistic populations (reviewed in O’Connor, 2012). A meta-analysis of an extensive experimental literature on early auditory evoked activity recorded with electro- or magneto-encephalography found group differences in the earliest auditory responses reflecting processing in the primary and secondary auditory cortices (Williams et al., 2021). Some aspects of speech perception, such as perception of speech in noise and attentional orienting to speech sounds, may be impaired in autistic individuals and might involve atypical hemispheric lateralization (Haesen et al., 2011; Jouravlev et al., 2020). Specific questions about mechanisms and heritability can be addressed by combining neurophysiological measurements of responses to controlled stimuli and including first-degree relatives in the study design.

Atypical auditory processing in early development may contribute to atypical development of higher-order functions relevant to autism. Auditory difficulties in adulthood may also have direct consequences on social, communicative, and rigid symptoms, for example, by engaging behavioral homeostatic mechanisms such as rigid adherence to routines or social avoidance to avoid perceptual overload. Self-reported noise sensitivity to a wide range of environmental sounds is considered a stable personality trait and is a significant predictor of individual adverse reactions to sounds (Ellermeier et al., 2020; Job, 1988). Noise sensitivity was also found to be correlated with the introversion dimension on the NEO Personality Inventory, which taps a construct that appears very similar to social QATs (Godoy-Gimenez et al., 2018; Shepherd et al., 2015). Similarly, extraversion on the Eysenck Personality Questionnaire was reported to be negatively correlated with noise annoyance (Belojevic et al., 2003; Dornic & Ekehammar, 1990). Thus, unusual auditory processing might be a transdiagnostic trait that contributes to disability rather than being specific to autism (Scheerer et al., 2021).

The study showed that individuals with tactile processing symptoms were more likely to have higher scores on social QATs (Figure 1(a) and Table 4), indicating decreased social motivation and enjoyment of social interactions. This aligns with previous research linking social introversion and social touch. For example, self-reported aversion to social touch was found to correlate positively with total QATs (Peled-Avron & Shamay-Tsoory, 2017; Ujiie & Takahashi, 2022; Voos et al., 2013), and parent-reported tactile hypersensitivity predicted an autism diagnosis in children (Jussila et al., 2020). Furthermore, avoidance of social touch was negatively correlated with extraversion on a personality inventory (Ujiie & Takahashi, 2022). The items on the tactile GSQ scale were designed to capture a range of differences common in autism, ranging from atypical pain, temperature, and touch processing, to disliking haircuts, clothes labels, or hugs. While these items load onto the same latent factor, at least with moderate reliability, it seems unlikely that differences in these domains are mediated by one mechanism. Tactile detection and discrimination thresholds might be altered in some autistic participants and might be related to excitation/inhibition imbalances, but findings have been mixed (Fukuyama et al., 2017; Sapey-Triomphe et al., 2019; Zetler et al., 2019). An affective touch functional magnetic resonance imaging paradigm (slow vs fast stroke) found negative correlations between QATs and blood oxygenation–level dependent responses in the superior temporal sulcus and orbitofrontal cortex, suggesting a role for C-tactile afferents and social brain networks (Voos et al., 2013). Another potential mechanism is atypical autonomic reactivity, which was found in response to touch in autistic adults with normal tactile thresholds (Fukuyama et al., 2017).

We found the proprioception subscale to be a stable and robust predictor of communication QATs across all analyses. It was the only modality–trait relationship that appeared to be as robust as associations involving auditory differences. The proprioception subscale of the GSQ comprises items concerning fine motor skills, interoceptive awareness, and perception of peripersonal space or body position, and thus probes a broader sensorimotor construct than its name suggests. The pragmatic language subscale of the BAPQ also contains behaviors that depend on motor skills (e.g. “I find it hard to get my words out smoothly” or “I speak too loudly or softly”) in addition to higher-order communication skills. Therefore, further studies could address whether the relationship is specific to motor function or reflects a broader association with higher language functions. In studies of infants, oral and fine motor skills have been found to predict later language capabilities (Belmonte et al., 2013; Iverson & Wozniak, 2007; LeBarton & Iverson, 2013; Leonard et al., 2015; Stevenson et al., 2017), suggesting that basic motor development is a prerequisite for the development of higher functions. This study preliminarily suggests that this association persists into adulthood. Consistent with this, a study on adults in a naturalistic conversation setting found that autistic participants demonstrated lower lexical diversity and produced fewer mouth movements (Parish-Morris et al., 2018). On a higher cognitive level, a neural overlap was found between syntactic processes and tool use in the basal ganglia, as well as a bidirectional cross-domain transfer of learning between these two skills (Thibault et al., 2021), raising the possibility of motor training to improve language development.

### Limitations

The main limitation of this study is the potential shortcomings of the GSQ in measuring sensory symptoms. While the GSQ is suitable for capturing autism-relevant sensory differences as well as measuring these in the general population (Kuiper et al., 2019; Robertson & Simmons, 2013; Sapey-Triomphe et al., 2018), the subscales contain only six items each and potentially included more than one neural construct. Some sensory-seeking behaviors are included in the GSQ but were under-represented in the auditory and gustatory modalities due to our removal of items that overlapped with rigid QATs. The moderate or low reliabilities of GSQ subscales also limit conclusions, especially in relation to negative findings. Measurement error variance has been shown to decrease the number of selected predictors by the SSVS (Bainter et al., 2020) and attenuate DA weights (Braun et al., 2019). This may have biased analyses, favoring variables with higher reliability in predicting QATs. For further studies on this topic, it will be critical to develop modality-specific instruments with better internal reliability, or to use objective measures of sensory functions. Thus, while our results did identify some sensory modalities as particularly important in explaining the known correlation between QATs and total sensory scores, they should not be used to motivate exclusion of other sensory modalities from further research.

Our use of anonymous data collection precluded clinical characterization of sensory differences or autistic traits, limiting generalizability beyond English-speaking adults with the cognitive resources to participate and access to the Internet. The dimensional individual differences approach is suitable for this experimental design as it does not rely on formal diagnoses, but it excludes lower-functioning subpopulations of autistic people who may have different patterns of sensory problems. The study relied on self-report of sensory differences and QATs, which has uncertain correspondence to functions that can be measured objectively in the laboratory, such as detection thresholds, attentional reorienting to stimuli, or autonomic reactivity (Fukuyama et al., 2017; Schulz & Stevenson, 2020, 2022; Yaguchi & Hidaka, 2020).

## Conclusion

This study suggests that sensory differences in autism are not fully generalizable across sensory modalities or QAT domains. Only auditory differences were robustly associated with all QAT domains, suggesting that they may have the strongest endophenotypic properties. The dominance of the auditory modality also supports the current consensus that auditory dysfunction is highly clinically relevant and closely associated with autism and subclinical autistic traits. Our findings also suggest that tactile dysfunction may specifically predict differences in social interaction, whereas proprioceptive or motor differences are more predictive of communicative QATs. In summary, we found that the relationship between sensory processing differences and endophenotypic QATs is at least, in part, modality-specific, encouraging in-depth studies on the mechanisms and intervention potential of neural processing within individual senses.
